# Simultaneous Thoracic Spine Metastatic Melanoma and Pre-existing Prostate Adenocarcinoma: A Unique Case Presentation and Literature Review

**DOI:** 10.7759/cureus.43429

**Published:** 2023-08-13

**Authors:** Srikanth A Venkata, Narek Hakobyan, Ruchi Yadav, Akriti Pokhrel, Fares Jamal, Omar Oudit, Avezbakiyev Boris, Arthur Kay

**Affiliations:** 1 Neurology, Brookdale University Hospital Medical Center, Brooklyn, USA; 2 Internal Medicine, Brookdale University Hospital Medical Center, Brooklyn, USA; 3 Hematology and Oncology, Brookdale University Hospital Medical Center, Brooklyn, USA; 4 College of Medicine, Saba University School of Medicine, The Bottom, BES; 5 Internal Medicine, Touro College of Osteopathic Medicine, Brooklyn, USA

**Keywords:** melanoma and nevi, prostate cancer (pca), melanoma primary, melanoma protocol, association: prostate cancer

## Abstract

In melanoma patients, distant metastases frequently manifest in the skin, lung, brain, liver, bone, and intestine. Notably, bone metastasis predominantly occurs within the axial skeleton, with the lumbar and thoracic spines being the most affected regions. Conversely, prostate cancer often disseminates to the bone, lung, liver, pleura, and adrenal glands. The spinal column, particularly the lumbar region, frequently harbors metastases in prostate cancer cases. Given the proximity of axial lesions to the spinal cord, patients commonly experience pain, weakness, and urinary dysfunction. This article presents a compelling case report of a patient initially diagnosed with metastatic prostate cancer, who later exhibited a metastatic lesion in the thoracic spine, subsequently identified as originating from acral melanoma on the plantar surface of the right foot. Histopathological examination confirmed the presence of acral melanoma in both the spine and the right foot. The patient received comprehensive treatment for advanced melanoma from a multidisciplinary team comprising medical and radiation oncologists. Considering the overlapping pathophysiology of prostate cancer and melanoma, simultaneous screening for both diseases in cases where one is detected could yield significant benefits, including enhanced morbidity and mortality outcomes and the facilitation of early detection for secondary malignancies.

## Introduction

Melanoma, characterized as one of the most lethal neoplasms [1], exhibits the highest level of aggressiveness among various forms of skin cancer [2]. Over the past decade, the incidence of melanoma has shown an upward trend [2]. Alarming statistics reveal that one in four patients diagnosed with melanoma succumb to the disease, while the five-year survival rate for metastatic melanoma stands at a mere 25% [2,3]. The American Cancer Society estimates that, in 2023, melanoma will claim the lives of 7,990 individuals in the United States alone [4]. Several risk factors contribute to the escalating incidence of melanoma, including exposure to ultraviolet light, family history, and clinical phenotype [2]. Notably, white individuals face a 20-fold higher likelihood of developing melanoma compared to their black counterparts, with an average age of diagnosis at 65 years [4]. Furthermore, melanoma exhibits the capacity to metastasize to diverse tissues, such as bone, brain, small bowel, and various other locations [5]. Solely 0.8% of melanoma cases manifest solitary metastasis to skeletal muscle, with bone metastasis constituting the sole site of metastasis in a mere 3.7% of melanoma diagnoses [5]. Notably, the advent of ipilimumab in 2011 and vemurafenib has led to a significant reduction in melanoma-related mortalities [2].

Prostate cancer ranks among the most prevalent malignancies [6], often causing urinary disturbances, bone pain, hematuria, and a range of additional symptoms [6]. Advanced age, race, family history, and obesity contribute to an increased risk of prostate cancer [6]. Metastasis of prostate cancer can occur in various locations, including the bones, leading to the formation of osteolytic lesions [6]. This paper focuses on a case involving a diagnosed melanoma that initially metastasized to the thoracic spine in a patient with a history of prostate cancer undergoing radiation therapy. 

## Case presentation

A 58-year-old male patient presented to the emergency department with complaints of generalized weakness and abdominal pain and a medical history significant for microcytic anemia, vitamin B12 deficiency, previous radiation therapy for prostate cancer, hyperlipidemia, and hypertension. He had a smoking history of 30 pack-years and is currently an active smoker. The patient experienced episodic, colicky upper abdominal pain that began three days prior to admission, accompanied by worsening constipation. Additionally, he reported mid-lower back pain without radiation to distant sites. Previously able to ambulate independently, he now had new-onset weakness in the bilateral lower extremities that limited his ability to perform daily activities, confining him to bed rest. The patient also mentioned experiencing urinary frequency, nocturia, and occasional dribbling over the past month.

**Figure 1:**
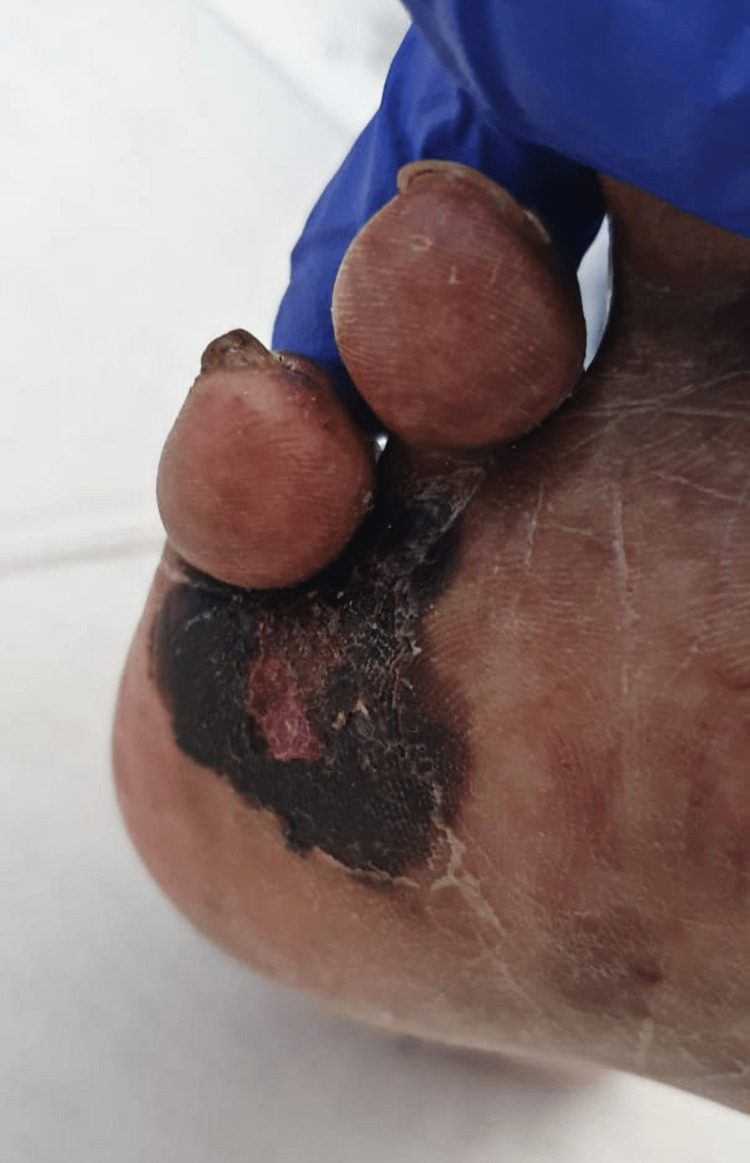
A clinical image illustrating a melanoma lesion on the right foot.

Upon admission, vital signs were within normal range, with a blood pressure of 154/63 mmHg, temperature of 37.1°C, pulse of 72 beats/min, respiratory rate of 19 breaths/min, and oxygen saturation of 96% on room air. Physical examination revealed the patient to be edentulous, with bilateral cataracts and a 12-centimeter left lower quadrant abdominal surgical scar from a Gridiron incision. Upper limb strength was intact bilaterally (5/5), while lower limb strength was diminished bilaterally (2/5). Notably, a black maculopapular lesion with irregular borders was observed on the plantar surface of the right foot (Figure 1). Due to suspected spinal cord compression, the patient was admitted, and neurology consultation resulted in the initiation of Decadron.

**Figure 2:**
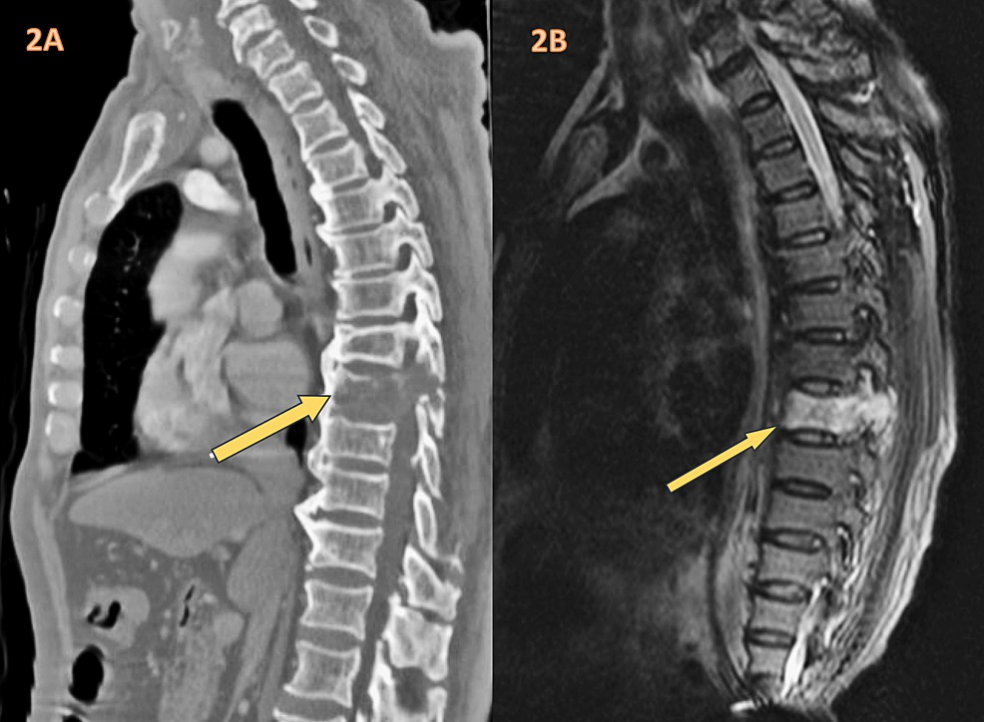
(A) Axial spine CT scan revealing metastatic lesion and (B) axial spine MRI displaying metastatic lesion. CT: computer tomography, MRI: magnetic resonance imaging.

Computer tomography (CT) imaging revealed nearly complete lytic replacement of the T8 vertebral body (Figure 2A). Further magnetic resonance imaging (MRI) investigations demonstrated numerous calvarial metastatic deposits, multifocal areas of heterogeneous T1, increased short tau inversion recovery signal in vertebral bodies and dorsal elements, as well as the lesion observed on T8 (Figure 2B). Mild right and left neural foraminal stenosis were also noted. Additionally, lumbar imaging displayed broad-based disc bulging with protrusions into the inferior neural foramina at L4-L5, accompanied by severe facet and ligament hypertrophy, nearly obliterating the central canal to a diameter of 2-3 mm. Mild to moderate neural foraminal stenosis was observed on both sides. At L5-S1, there was broad-based disc bulging with facet and ligamentous hypertrophy.

To decompress the spinal canal, a T8 laminectomy was recommended, during which a biopsy of the epidural mass was obtained. Simultaneously, a biopsy of the hyperpigmented lesion on the plantar surface of the right foot was performed. The epidural biopsy exhibited findings consistent with malignant melanoma, while the foot lesion biopsy showed dermal segments also consistent with malignant melanocyte presence. A diagnosis of metastatic acral melanoma was established, and a liquid biopsy was sent for confirmation of cancer.

## Discussion

Skin accounts for the majority (90%) of primary melanoma sites, whereas only a small proportion involves mucous membranes and eyes [7]. Certain cases of melanoma with an unknown primary lesion can metastasize to the spinal cord, leading to the presentation of radiculopathy [8]. Initially, patients with melanoma metastasis to the bone may remain asymptomatic; however, as the disease progresses, they may experience bone pain, fractures, spinal cord compression, and cachexia [7]. The primary treatment modality for melanoma involves surgical excision of the tumor with appropriate margins [9]. Sentinel lymph node biopsy is recommended for tumors measuring 0.8 mm or thicker or in the presence of ulceration [9]. Surgical interventions for metastatic melanoma are not curative; therefore, they are typically combined with chemotherapy or immunotherapy approaches [9].

Conversely, the bone serves as the most frequent site for prostate cancer metastasis, followed by the lungs, liver, pleura, and adrenal glands [10]. When prostate cancer metastasizes to the bones, the spine is the most affected site, with rare occurrences of metastasis to long bones, ribs, or the skull [10]. Treatment strategies for prostate cancer depend on the individual case and may include observation and surveillance, prostatectomy, radiation therapy, or other tailored approaches [11].

Melanoma ranks as the fifth most prevalent cancer in the United States [12]. The multifactorial nature of melanoma pathogenesis involves a combination of genetic and environmental factors that contribute to increased risk [12]. Conversely, prostate cancer holds the distinction of being the most common malignancy among males in the United States and the second leading cause of death [12]. Androgen deprivation therapy serves as the primary treatment modality for prostate cancer [13]. Previous reports in 2011 and 2012 indicated an association between prostate cancer and an elevated risk of developing melanoma [12]. Melanocytes possess the capability to convert androgens into dihydrotestosterone, which potentially underlies the link between prostate cancer and melanoma [14]. Notably, melanocytes exhibit high susceptibility to androgen influences [12]. A study conducted by Allil et al. demonstrated an increased melanoma risk in mice injected with testosterone [15]. Similarly, a study by Li et al. involving 42,372 individuals revealed a significant elevated risk of melanoma among prostate cancer patients, with a hazard ratio of 1.83 (95% confidence interval 1.32-2.54) [16]. This association can be attributed to the shared pathogenic factors involving androgens in prostate cancer and melanoma [16]. In the Utah population, individuals with relatives diagnosed with melanoma displayed an increased risk of prostate cancer, and vice versa [14]. Moreover, analysis of a Swedish family cancer database, encompassing 15.7 million individuals, indicated that families with a history of three prostate cancer cases had twice the risk of developing melanoma [14]. In a case-control study conducted by Goldenberg et al., prostate cancer patients with melanoma exhibited a higher odds ratio compared to those without melanoma [12]. Androgens stimulate telomerase activity, promoting telomere elongation and thereby increasing the risk of melanoma [12]. Furthermore, androgens may augment melanoma risk by suppressing the immune system [17]. Hsueh et al. reported improved survival in melanoma patients upon blocking androgen signaling [17]. Additionally, Sutcliffe et al. conducted a study illustrating an increased risk of prostate cancer associated with severe acne, an inflammatory condition driven by androgens [18].

Numerous studies have explored the correlation between melanoma and prostate cancer, suggesting a potential predisposing state in which prostate cancer is more likely to occur in individuals diagnosed with melanoma [19]. These investigations align with long-standing hypotheses regarding the relationship between these two cancer types, focusing on their relative frequencies and temporal co-occurrences in patients. Metanalytical studies have reported a statistically significant association, indicating that 6% of men diagnosed with melanoma in their sample size (n=1899) subsequently developed prostate cancer, compared to 4% of men without a melanoma diagnosis who developed prostate cancer [19]. Notably, these findings also demonstrated a significant relationship between the interval between the initial melanoma diagnosis and the subsequent development of prostate cancer [19]. Specifically, men with a melanoma diagnosis more than ten to fifteen years prior exhibited a higher likelihood of developing prostate cancer than those diagnosed within one to ten years [19]. These findings support the notion of an association between the development of prostate cancer and prior melanoma diagnoses, warranting further investigation into the potential utility of prostate cancer screening in patients with a history of melanoma. Early detection and treatment of prostate cancer in this patient population may be facilitated by recognizing this relationship [19]. It is important to acknowledge that this association is not unidirectional. Some studies have indicated a reciprocal risk, demonstrating an increased likelihood of developing melanoma in patients diagnosed with prostate cancer who exhibit long-term survival [16,19].

Multiple studies have been conducted to investigate the pathological mechanisms underlying melanoma transformation and malignancy, revealing genetic loci that contribute to the malignant properties of melanoma cells [20-22]. One such locus is PTEN, which encodes the phosphatase and tensin homolog protein. PTEN mutations are observed in various human malignancies, including lung cancer, breast cancer, glioblastomas, melanomas, and prostate cancer [21]. PTEN acts as a tumor suppressor by regulating the cell cycle to prevent excessive cellular division, mutation accumulation, and subsequent malignant transformation [21]. The PTEN protein possesses two primary biochemical functions: lipid phosphatase and protein phosphatase activities [20]. The lipid phosphatase activity inhibits the conversion of phosphatidylinositol 4,5 bisphosphate (PIP2) to phosphatidylinositol 3,4,5 trisphosphate (PIP3), thereby reducing downstream Akt levels [20]. Akt protein activity plays a role in cell cycle regulation, cell survival, and oncogenic activity through uncontrolled cellular division [20-23]. PTEN also upregulates the cyclin-dependent kinase inhibitor p27, which arrests the cell cycle at the G1/S phase, and it modulates proapoptotic and antiapoptotic signaling molecules, thereby influencing tumorigenesis [20]. In contrast, the protein phosphatase activity of PTEN is less involved in conferring transformative capacities to melanoma cells but is implicated in inhibiting aberrant cell dispersion, promoting cellular adhesion, restraining rapid growth, and preventing evasion of cellular apoptosis [21-23]. The combined functions of PTEN provide protective tumor-suppressive effects, and loss-of-function mutations in this locus increase the risk of developing melanoma and prostate cancer. Various studies have demonstrated a graded increase in the risk of developing these malignancies with dose-dependent PTEN functional loss [20]. Molecular analysis of prostate malignancies at different stages has revealed the upregulation of multiple cell signaling pathways related to cell survival and growth upon PTEN loss-of-function [20]. Furthermore, mouse models have demonstrated that PTEN loss-of-function and subsequent upregulation of prosurvival pathways lead to the metastatic stage of prostate cancer [21]. These findings support the notion that the absence of PTEN function plays a shared tumorigenic role in both melanoma and prostate cancer. Therefore, when patients present with either of these malignancies, further investigation for the other cancer form should be considered due to their shared molecular pathology [22-25].

The US Preventive Service Task Force (USPSTF) does not currently find sufficient evidence to support screening for melanoma, while the Canadian Cancer Society and Australian Cancer Network recommend screening for populations at high risk [26-28]. Physicians who suspect melanoma should apply the ABCDE rule, which involves assessing the lesion for asymmetry, irregular borders, inconsistent color, diameter greater than 6 mm, and evolution over time [29]. Additionally, further investigation should be conducted when there is a lesion that appears different from the others, known as the "ugly duckling sign," or when other alarming features are present, such as a lesion beneath a nail, bleeding, or itching [29]. Genetic testing has shown increased detection of Melanoma in patients with multiple family members diagnosed with the disease [30]. High-risk patients, including white adults over 50 years old, those with large atypical nevi or more than 50 nevi, individuals with a history of skin cancer, those on immunosuppression medications, those with extreme sun sensitivity or red hair, and individuals with a strong family history of melanoma (at least two second-degree relatives from the same side or one or more first-degree relatives, while those with more than three relatives from the same side are considered very high risk), should undergo annual whole-body screenings performed by physicians trained in skin examination [30]. However, according to the USPSTF, the Australian Cancer Network, and the Cancer Council Australia, routine screening for melanoma is not recommended for patients who are not considered high-risk [26,27,31]. Non-high-risk patients should still receive screening for melanoma during their routine visits, particularly in areas that are difficult for them to self-examine [30]. It is important to note that patients with darker skin are at an increased risk for Acral melanoma, and therefore a focused examination of their digits, soles, and palms should be conducted during routine visits [30].

Early screening plays a crucial role not only in detecting the disease but also in reducing morbidity and mortality and improving the quality of life for patients. A study by Jahn et al. demonstrated an increase in the incidence of prostate cancer following the use of prostatic specific antigen (PSA) as a screening method, but the incidence returned to baseline after a decrease in PSA testing [32]. Another study conducted by Ilic et al., which involved 162,243 men aged between 50 and 69 years with a median follow-up of 12 years, revealed a lower incidence of metastatic prostate cancer in regularly screened patients compared to those who were not screened [33]. However, a study by Etzioni et al. showed that screening for prostate cancer did not significantly decrease mortality [34].

In the study conducted by Schröder et al., the mortality rate in the screened group was lower than in the control group, with a rate of 0.53/1000 men in the screened group compared to 0.66/1000 men in the control group [35]. However, a large study involving 76,774 men did not show a mortality benefit from screening [36]. Moreover, screening did not demonstrate an increase in the quality of life for patients, which is a significant aspect of the importance of screening [37]. When a patient has an elevated PSA level, a prostate biopsy is typically performed [38]. Prostate biopsy is associated with various complications, including pain, bleeding, infection, and urinary obstruction [38]. Overdiagnosis of prostate cancer poses a major challenge in screening as it may lead to unnecessary treatment for a clinically insignificant cancer [37]. Treatment for prostate cancer carries a range of complications, such as urinary incontinence and impotence [37]. These complications could be avoided through surveillance for patients with prostate cancer rather than active treatment [39]. Additionally, the risk of overdiagnosis increases with age [40]. Certain factors, such as age over 50, breast cancer gene (BRCA) mutations, MSH2 and MSH6 mutations, family history, and being of Black ethnicity, can elevate the risk of prostate cancer [37].

Based on a comprehensive review of existing data on melanoma and prostate cancer, no specific recommendation has been established regarding screening for one disease in the presence of the other. While an association between prostate cancer and melanoma has been discussed earlier, there is currently no specific diagnostic test recommended for detecting melanoma in prostate cancer patients or vice versa.

## Conclusions

Due to the intricate interconnection and shared pathophysiology between prostate cancer and melanoma, we foresee that if either of these diseases is detected in a patient, thorough screening for the other could be of immense importance. This proactive approach not only holds the potential to enhance overall morbidity and mortality outcomes but also enables the early detection of secondary malignancies. By recognizing the underlying associations and molecular pathways involved in both cancers, clinicians can adopt a comprehensive screening strategy that addresses the unique risks and characteristics associated with each disease. The simultaneous evaluation of prostate cancer and melanoma in affected individuals can lead to timely interventions, personalized treatment plans, and improved patient outcomes. Furthermore, such an integrated screening approach may unveil common genetic predispositions and shared risk factors that could help guide targeted prevention strategies and potentially uncover novel therapeutic avenues for both diseases. Therefore, considering the intricate relationship between prostate cancer and melanoma, it is imperative to implement screening protocols that encompass both conditions, facilitating a holistic approach to cancer management and ultimately benefiting patient care.
